# Neuroendocrine cells of the prostate: Histology, biological functions, and molecular mechanisms

**DOI:** 10.1093/pcmedi/pbab003

**Published:** 2021-01-28

**Authors:** William Butler, Jiaoti Huang

**Affiliations:** Department of Pathology, Duke University School of Medicine, Durham, NC 27710, USA; Department of Pathology, Duke University School of Medicine, Durham, NC 27710, USA

**Keywords:** prostate cancer, neuroendocrine

## Abstract

Prostate cancer (PCa) is a common cause of cancer-related mortality in men worldwide. Although most men are diagnosed with low grade, indolent tumors that are potentially curable, a significant subset develops advanced disease where hormone therapy is required to target the androgen receptor (AR). Despite its initial effect, hormone therapy eventually fails and the tumor progresses to lethal stages even through continued inhibition of AR. This review article focuses on the role of PCa cellular heterogeneity in therapy resistance and disease progression. Although AR-positive luminal-type cells represent the vast majority of PCa cells, there exists a minor component of AR-negative neuroendocrine (NE) cells that are resistant to hormonal therapy and are enriched by the treatment. In addition, it is now well accepted that a significant subset of hormonally treated tumors recur as small cell neuroendocrine carcinoma (SCNC), further highlighting the importance of targeting NE cells in addition to the more abundant luminal-type cancer cells. Although it has been long recognized that NE cells are present in PCa, their underlying function in benign prostate and molecular mechanisms contributing to PCa progression remains poorly understood. In this article, we review the morphology and function of NE cells in benign prostate and PCa as well as underlying molecular mechanisms. In addition, we review the major reported mechanisms for transformation from common adenocarcinoma histology to the highly lethal SCNC, a significant clinical challenge in the management of advanced PCa.

## Introduction

Prostate cancer (PCa) is the most common non-cutaneous malignancy and second leading cause of cancer-related mortality in men.^1^ As a result of widespread prostate-specific antigen (PSA) screening, the vast majority of PCa cases are detected in early, asymptomatic stages of disease where treatment options include active surveillance, radical prostatectomy, and radiation.^2^, ^3^ Although most cases are cured with local therapy, a significant subset of men develop biochemical recurrence followed by metastasis, for whom systemic treatment becomes the only option.^4^ As androgen receptor (AR) signaling is critical for the proliferation and metabolic functions of prostatic tumor cells,^5^ the mainstay of treatment is hormonal therapy, aimed at reducing the systemic level of circulating androgens (i.e. GnRH agonists and antagonists), inhibiting intratumoral androgen synthesis (i.e. abiraterone acetate) or competitive inhibition of AR itself (i.e. enzalutamide). This treatment, although initially effective, eventually fails after the tumor cells develop various mechanisms of resistance. The diseases at this stage are known as castration-resistant prostate cancer (CRPC), an advanced form of the disease with a median survival of 9–36 months. Research has been ongoing for many years to understand the major mechanisms as to how prostate tumor cells become resistant to AR-targeted therapies as well as methods to circumvent this resistance.^6–10^ Unfortunately, agents that further inhibit AR have not significantly improved patient survival and a growing theme has evolved to search for AR-independent strategies to target PCa.

PCa exhibits cellular heterogeneity which may have a significant role in pathology and resistance to hormonal therapy. In benign prostate, luminal (also known as secretory cells) and basal cells comprise the major epithelial compartments. However, there exists a minor component of epithelial cells possessing neuroendocrine (NE) features, representing only ∼1% of the entire epithelial cell population.^11–13^ The vast majority of PCa cases are histologically classified as adenocarcinoma characterized by loss of basal cells and proliferation of malignant luminal-type cells expressing AR and PSA. Importantly, every case of prostate adenocarcinoma also contains a minor component of NE cells that are negative for AR and PSA.^13^, ^14^ Because NE cells are AR negative and androgen-independent, they are spared by hormonal therapy and subsequently, enriched in the tumor.^15^, ^16^ Furthermore, 17%–30% of recurrent castration-resistant tumors display a variant histology known as small cell neuroendocrine carcinoma (SCNC)^17^, ^18^ with the tumor composed entirely of NE cells. SCNC is resistant to traditional hormonal therapy and carries the poorest prognosis of all cancers of the prostate.^19^, ^20^ It is therefore hypothesized that to achieve maximal therapeutic efficacy, the NE cells must be targeted as well. This review provides a modern perspective on the role of NE cells in PCa, including their various histologic contexts, biological roles, and molecular mechanisms. Furthermore, current theories on the development of SCNC histology from adenocarcinoma after hormonal therapy are discussed.

## Histologic context of neuroendocrine cells in prostate cancer

NE cells are present in benign prostate and throughout the entire spectrum of PCa.^11^, ^13^ In benign prostate, they act as sensors to various stimuli and secrete neuropeptides and cytokines to maintain the surrounding epithelial population.^11^, ^21^ Their function in PCa varies depending on their histological context but it is generally understood that the pathogenicity of NE cells increases as PCa progresses. This section reviews the common histological settings of NE cells as well as the most commonly used immunohistochemical markers for highlighting them in the epithelium.

### Cellular heterogeneity of the human prostate

The prostate is an epithelial organ, composed of glandular structures surrounded by a fibromuscular stroma containing blood vessels and nerves. Glands in the benign prostate are often large and irregularly shaped, lined by a stratified cuboidal to columnar epithelium.^22^ The layer of cells bordering the lumen are commonly termed the *luminal (or secretory) cells*. These cells are identified easily on standard hematoxylin and eosin (H&E) stained slides and contain abundant clear to eosinophilic cytoplasm and rounded nuclei towards the basal aspect of the cell.^22^ They are terminally differentiated and possess secretory functions. Commonly used immunohistochemical markers for luminal cells are AR, PSA, NKX3.1, TMPRSS2, and cytokeratins 8 and 18.^23^ The outer layer of cells having direct contact with stroma are termed the *basal cells*, and are commonly thought to be the progenitor cells of the prostate with the ability to differentiate into secretory populations. These cells are actively proliferating and contain more ovoid nuclei and less cytoplasm compared with luminal cells. They can be identified using standard H&E staining as well as through the use of immunohistochemical markers p63 and high molecular weight cytokeratins (34βE12, and cytokeratins 5/6 and 14).^22^, ^23^

**Figure 1.**
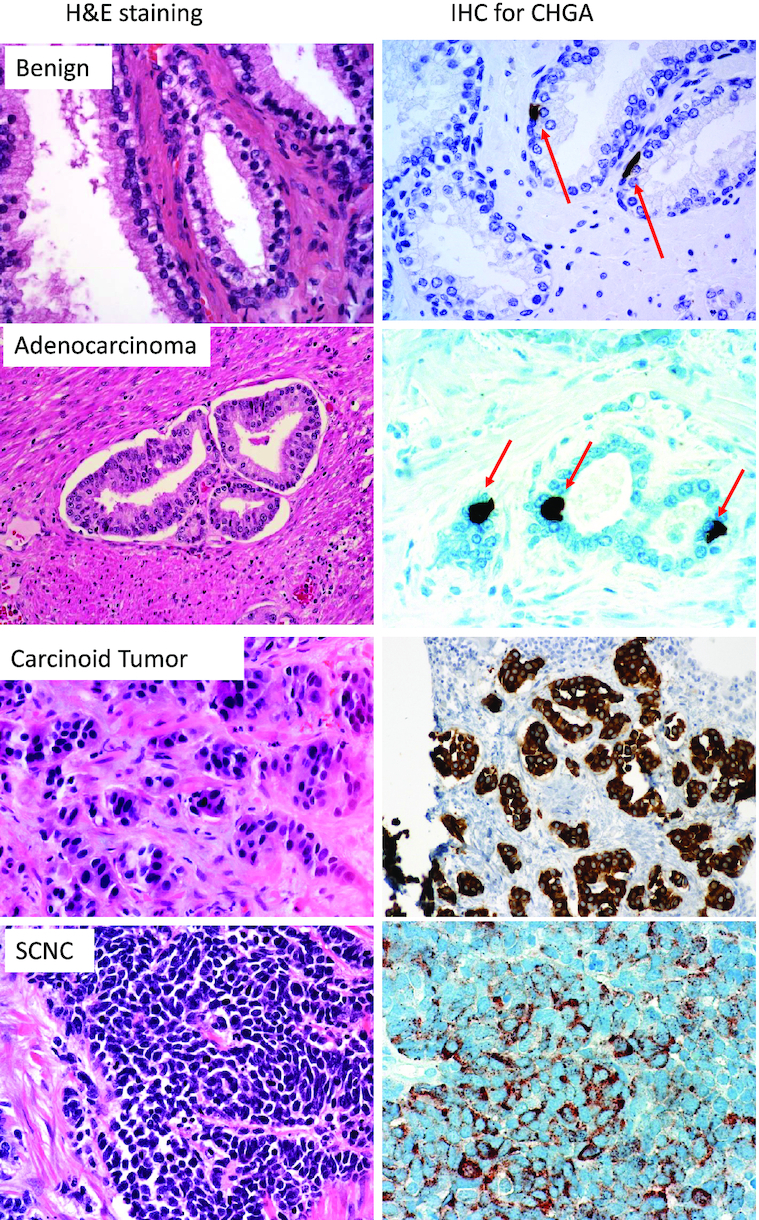
The left panel shows H&E stained images of benign prostate, untreated adenocarcinoma, carcinoid tumor, and SCNC (from top to bottom). The right panel shows immunohistochemical staining for the expression of NE marker CHGA in each tumor type. There are rare NE cells in benign prostate and adenocarcinoma while carcinoid tumor and SCNC express CHGA diffusely.

NE cells are scattered among the more abundant luminal and basal cells, representing only ∼1% of the epithelial population.^11^, ^13^, ^21^ Because of their rarity in benign prostate and most prostate tumors, they cannot be appreciated on standard H&E stained sections. However, through the use of electron microscopy, they can be identified and differentiated from luminal and basal cells by their elongated cell bodies and intracytoplasmic dense core secretory granules.^21^ Furthermore, electron microscopy can differentiate between two morphologic subtypes of NE cells: open-type and closed-type.^24^ Open-type cells possess long microvilli which reach the lumen and can detect changes in pH as well as respond to other chemical stimuli in luminal secretions. Closed-type cells possess dendritic-like processes and receive stimuli from nerve endings, blood vessels, and smooth muscle cells. A more practical way to highlight NE cells in the prostatic epithelium involves the use of immunohistochemistry (IHC) (Fig. 1) and commonly used markers include chromogranin A (CgA),^11^, ^13^, ^21^ synaptophysin (SYP),^11^, ^13^, ^21^ neuron-specific enolase (NSE),^11^, ^13^, ^21^ neural cell adhesion molecule (NCAM or CD56),^11^, ^13^, ^21^ forkhead-box A2 (FOXA2),^25^ and CXC chemokine receptor 2 (CXCR2).^26^ In addition to being negative for AR and PSA, they are also negative for Ki67 because they are non-proliferative, post-mitotic cells.^11^, ^13^, ^21^, ^26^

### Neuroendocrine cells in prostatic adenocarcinoma

It is not routine to perform IHC for NE markers in the setting of non-hormonally treated adenocarcinomas as their presence and quantity would not affect the choice of treatment. However, an association between the number of NE cells in hormone-naïve tumors and progression to CRPC has been found and deserves to be further studied.^27^ Although it can vary among patients, NE cells usually make up ∼1% of tumor cells in a hormone-naïve setting (Fig. 1). In tumors that have been treated with hormone therapy, NE cells commonly become enriched to ∼5%–10% of the tumor cell population.^26^, ^28^ In these cases, NE cells commonly appear as clustered foci and the World Health Organization (WHO) has classified this pathology as “focal neuroendocrine differentiation (NED) in conventional prostatic adenocarcinoma”.^29^ Currently, this finding is highly variable among patients and has not been found to increase the likelihood of developing SCNC. Occasionally, NE cells in this setting will demonstrate intense eosinophilia with large cytoplasmic granules and this morphology has been classified as “adenocarcinoma with Paneth cell NED”,^29^ which generally pursues an indolent course.

### Small cell neuroendocrine carcinoma

Although adenocarcinoma containing rare NE cells represents the most common initial presentation, rare primary PCas (∼1%) are histologically classified as SCNC, composed entirely of NE tumor cells.^30^ As the tumor cells do not secrete PSA, they are not detected by traditional screening methods and are often disseminated at presentation carrying a poor prognosis with a median survival of < 2 years.^19^, ^20^ More commonly, SCNC is detected as a recurrent tumor in patients following hormone therapy for prostatic adenocarcinoma.^31^ This was once thought to be a rare presentation; however, a recent multi-institutional study revealed that treatment-induced small cell carcinoma (t-SCNC) comprises 17%–30% of clinical CRPC.^18^, ^32^ As with primary SCNC, these tumors carry a high mortality rate and there has been a recent surge in studies seeking to differentiate the genetic landscape between CRPC-adenocarcinoma and t-SCNC tumors to find better treatment modalities. Histologically, SCNC is characterized by a high nuclear/cytoplasmic ratio, scanty cytoplasm, hyperchromatic nuclei, nuclear molding, frequent mitotic figures, and necrosis.^33^, ^34^ Unlike NE cells in benign prostate and adenocarcinoma, NE tumor cells in SCNC are highly proliferative with a high Ki67 labeling index.^33^, ^34^ Pathologic diagnosis based on histological features is considered the gold standard. IHC staining can be helpful in difficult cases as one or more NE markers are positive in ∼90% of cases (Fig. 1).^33^ Pure SCNC is uncommon, as in most cases SCNC is mixed with adenocarcinoma components. In these mixed SCNC-adenocarcinoma cases, the SCNC component can have a wide range and the Gleason grade of the adenocarcinoma component is almost always ≥ 8.^29^ The transition between the two morphologies is often abrupt and easily recognizable without the use of IHC.

SCNC represents the most common presentation of neuroendocrine PCa. However, there are two other rare tumor types also composed entirely of NE cells: carcinoid tumor and large cell neuroendocrine carcinoma (LCNC).^29^, ^35^, ^36^ Carcinoid tumors are extremely rare and appear as well-differentiated organized nests of uniform tumor cells that are positive for NE markers (Fig. 1) and are not associated with prostatic adenocarcinoma. Although carcinoid tumors in other organs are relatively indolent, their behavior as a primary prostate tumor is uncertain because of their rarity, with only a few cases reported.^37–40^ LCNC is also exceptionally rare and can be differentiated from SCNC by larger amounts of cytoplasm, more prominent nucleoli, and peripheral palisading of tumor foci.^29^, ^35^, ^36^ These tumors were once thought to almost always occur following hormone therapy based on the 7 patients followed by Evans *et al*.^41^; however, a recent pooled analysis of 20 patients showed that 9/20 (45%) were reported as *de novo* with only 8/20 (40%) having a history of hormone therapy for prostatic adenocarcinoma.^42^ All patients with LCNC displayed rapid deterioration and death and the median survival is thought to be 7 months.

**Figure 2.**
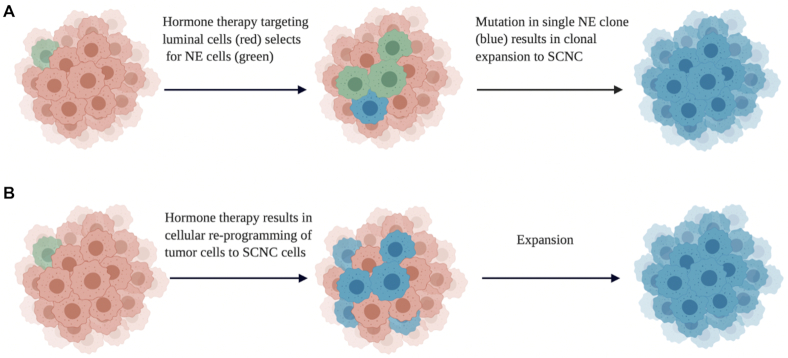
Current theories of SCNC development from adenocarcinoma. (A) Clonal expansion of pre-existing NE cells in adenocarcinoma followed by mutation and expansion. (B) Trans-differentiation of luminal cell clones to SCNC followed by expansion (Created with BioRender.com).

In comparison with carcinoid tumor and LCNC that are exceedingly rare, SCNC has become an increasingly recognized clinical concern in the management of patients with advanced PCa because of its high prevalence following hormonal therapy. The molecular and cellular basis of the phenotypic transition from a tumor traditionally displaying adenocarcinoma features to SCNC still needs more research. Importantly, it is unknown whether SCNC arises by clonal expansion of pre-existing NE cells in adenocarcinoma or trans-differentiation of luminal-type cells as a mechanism to evade hormonal therapy (Fig. 2).

## Origin and biological function of neuroendocrine cells

NE cells are present in benign and malignant glands of the prostate, representing ∼1% of the epithelial cell population. They are considered terminally differentiated distributed in all anatomic zones, with higher concentrations in the transitional and peripheral zones compared with the central zone.^43^ The cellular origin of NE cells has not been firmly established. It has been hypothesized that NE cells may arise in the neural crest and migrate to the prostatic epithelium because of the observed appearance of CgA + cells in the paraganglia of future prostatic mesenchyme and eventual dispersion in urogenital mesenchyme.^44^ With the sprouting and development of prostatic analgen from the urogenital epithelium, it is thought that NE cells migrate to the basal layer of the mature glands where they are then commonly observed.^44^ However, more recent studies have shown strong evidence that NE cells are the progeny of basal cells as NE cells express basal-specific keratins and their differentiation from human pluripotent (c-kit+) basal cells has been well demonstrated *in vitro*.^45^, ^46^ In an *in vivo* study, Goldstein *et al*. showed that primary benign human prostate basal cells can be transformed into acinar-type adenocarcinoma-containing NE cells, providing strong evidence that NE cells may be derived from benign basal cells of the prostate.^47^

The exact function of NE cells has not been well elucidated because of the rarity of the cell population and most hypotheses have been largely based on immunostaining patterns. For example, it is known that NE cells have a secretory function as they express various peptide hormones including neural growth factor (NGF), bombesin/gastrin-releasing peptide, serotonin, histamine, calcitonin, neuropeptide Y, vasoactive intestinal peptide, parathyroid hormone-related protein, somatostatin, and vascular endothelial growth factor.^11^, ^21^, ^48^ Although they are considered quiescent, non-proliferative cells, they may communicate with nearby epithelial cells in a paracrine manner.^11^, ^21^ This cross-talk is evidenced by the observation that luminal cells express receptors for many NE products. For example, receptors for serotonin (5HT1a),^49^ bombesin (GRPR),^50^ neurotensin,^51^ somatostatin (SSTR1–5),^52–54^ neuropeptide Y,^55^ and calcitonin,^56^ have all been detected in benign prostate and human prostate PCa. In addition, it has been shown by our group that NE cells secrete interleukin-8 (IL-8), while luminal cells express IL-8 receptor-type 1 (CXCR1).^26^ In subsequent studies, it was shown that IL-8 induces androgen-independent proliferation of PCa cells and that this may result from modulation of AR levels and activity.^57^, ^58^ It has also been shown that NE-cell product serotonin (5-HT) influences the growth of luminal cells by AR modulation, further highlighting the importance of cross-talk between NE cells and the surrounding luminal-type tumor cells in maintaining the growth of the entire epithelial cell population.^59^ As luminal cells respond to hormonal therapy while NE cells do not, it is reasonable to hypothesize that strategies to specifically target NE cells, alone or in combination with AR targeting, hold the key to long-term disease control or cure. A successful example of such a strategy was recently published by our group showing that selectively targeting NE cells using navarixin, a CXCR2 inhibitor, can suppress the growth of PCa cells both *in vitro* and in xenograft models.^60^

## Molecular mechanisms of neuroendocrine differentiation in prostate cancer

NE cells likely play important roles throughout the entire disease spectrum of PCa including CRPC-adenocarcinoma and SCNC. As patients initially diagnosed with adenocarcinoma can recur with SCNC,^18^, ^32^ significant research efforts have focused on elucidating the mechanisms leading to the development of SCNC following hormonal therapy. There is considerable debate in the field as to whether SCNC arises by clonal expansion of pre-existing NE cells in adenocarcinoma or trans-differentiation of luminal cells through lineage plasticity (Fig. 2). In addition, as various stimuli have been noted to induce NED *in vitro*, it has been challenging for translational scientists to identify which stimuli and drivers are clinically relevant in humans. This section reviews the major signaling and genomic drivers of NED in PCa as currently reported in the literature.

### Intracellular signaling and neuroendocrine differentiation

As early as 1999, it was observed that LNCaP cells, modeling hormone-naïve adenocarcinoma, expressed NE markers following culture in androgen-deprived medium and that this phenotype was reversible after the re-addition of androgen.^61^, ^62^ This has become a well-accepted model for studying the dynamics of NED and exploring the signaling events that occur in the process. For example, it has been found that ERK/MAPK activity is activated in LNCaP cells that have been treated with androgen withdrawal and that use of the MEK inhibitor, PD8059, effectively inhibited ERK and reversed the acquired NE characteristics.^63^, ^64^ It was further shown that ERK/MAPK activity in this context is driven by IL-8 secretion mediated by FOXA1 loss.^65^ In addition to the MAPK pathway, several groups found that cells increase their cyclic-AMP levels during NED.^66–68^ This has been found to lead to increased cAMP-dependent protein kinase A (PKA) activity,^67^, ^69^ leading to the expression of NE markers, secretion of neuropeptides, and low mitotic activity (characteristic of their quiescent nature in an adenocarcinoma setting). The mechanism as to how cAMP/PKA leads to NE phenotype has been proposed as downstream expression of cAMP-responsive binding element, CREB, which following androgen withdrawal, up-regulates the G-protein coupled receptor, GRK3.^70^ Knockdown of GRK3 led to complete reversal of the cAMP/PKA-induced phenotype; however, the direct mechanism as to how GRK3 mediates NE phenotype is largely unknown. Furthermore, PKA signaling has been shown to inhibit anti-proliferative factors, including Ras homolog gene family member A (RhoA), and Rho-associated coiled-coil containing protein kinase (ROCK).^71^ In addition to cell survival mechanisms, it has been noted that NE cells are a significant source of vascular-endothelial growth factor-A (VEGF-A).^72^ Angiogenesis is believed to have a significant role in CRPC progression and several clinical trials have attempted targeting VEGF-A in combination with hormone therapy, although mixed results have been obtained as to the efficacy of this treatment.^73^

Finally, it has been shown by our group that NE cells express CXCR2 and its native ligand, IL-8.^26^ It has been suggested that the growth inhibitory IL-8-CXCR2-p53 cascade maintains NE cells in a quiescent state and that following p53 mutation, the growth inhibitory signaling is lost leading to lethal SCNC.^74^ It has also been shown that CXCR2 overexpression in LNCaP cells drives a neuroendocrine phenotype, and this may be a result of up-regulated PI3K/AKT, MAPK, and VEGFR signaling pathways.^60^ Further studies are needed to fully understand the important role of CXCR2 in altering intracellular signaling for the emergence of NE phenotype.

### Genomic and molecular hallmarks of small cell neuroendocrine carcinoma

Over the past 10 years, the genomic landscape of human SCNC has become more illuminated through several well-resourced studies with valuable patient cohorts. It is well-accepted that human SCNC harbors frequent mutations of TP53 and Rb1.^17^, ^74^, ^75^ As Rb1 is a critical regulator of the cell cycle and p53 is a critical regulator of apoptosis, inactivation of these genes leads to the hyperproliferative, anti-apoptotic phenotype observed in SCNC. Furthermore, it has been shown *in vitro* that Rb1/p53 deletion in hormone-resistant PCa cells (LNCaP/AR) causes SCNC phenotype and that this observed lineage plasticity is caused by up-regulation of the reprogramming factors, SOX2 and EZH2.^76^, ^77^ In addition to p53 and Rb1 lesions, it has been observed that the oncogene MYCN and Aurora-Kinase A (AURKA) are frequently overexpressed in SCNC compared with CRPC-adenocarcinoma.^32^ AURKA has roles in mitosis and cell cycle regulation but has also been shown to stabilize MYCN, suggesting a possible collaborative role for the two proteins.^78^ In addition, MYCN is capable of promoting NED, and this may be mediated in part by EZH2.^79^

Several additional transcription factors to MYCN and SOX2 have been associated with NE phenotype, including ASCL1,^80^ FOXA2,^25^ E2F1,^81^ ONECUT2,^82^ REST,^83^ and BRN2.^84^ In particular, FOXA2, ONECUT2, REST, and BRN2 were observed to drive NE phenotype from models displaying classical adenocarcinoma features. For example, it was observed in TRAMP mice that HIF-1α, regulated upstream by Siah2, forms a complex with FOXA2 and activates a transcriptional program required for NE tumor development.^85^ ONECUT2 was found to also regulate HIF-1α gene signature to promote NE phenotype,^82^ in addition to other mechanisms such as suppression of AR signaling as well as blocking the expression of the luminal-defining transcription factor, FOXA1.^86^ REST, a transcriptional repressor of neuronal differentiation, becomes down-regulated in the progression to SCNC with concomitant up-regulation of neuronal genes.^83^ Subsequently, it was discovered that alternative splicing by SRRM4, a master regulator of transdifferentiation of embryonic stem cells to neural cells, is responsible for lower REST transcript and higher levels of the truncated transcript, REST4.^87^ This mechanism was demonstrated *in vitro* and it has been shown in human SCNC specimens that SRRM4 is negatively associated with REST/REST4 ratio. Furthermore, it has been shown that AR inhibition blocks the translation of REST protein, producing an additive effect to the SRRM4-mediated pathway. BRN2, a master regulator of neural differentiation, has been shown to be suppressed by AR, upregulated in NE tumors, and important in maintaining the NE phenotype via direct regulation of the epigenetic re-programmer, SOX2.^84^ Collectively, these findings demonstrate the important role transcription factors play in re-programming cells to undergo NED as well as maintaining the phenotype. Further studies to understand the relationships among these many transcription factors are needed to further understand the complex biology of NED.

Several other oncogenic drivers and mechanisms of SCNC genesis have been reported. In patient-derived xenograft systems modeling the progression of prostatic adenocarcinoma to SCNC, it was observed that the placental gene PEG10 becomes de-repressed and highly up-regulated.^88^ It was found that both AR and E2F/Rb pathways dynamically regulate distinct isoforms of PEG10 at different stages of SCNC development and that PEG10 was able to drive cell cycle progression in the context of p53 loss as well as promote invasion through up-regulated Snail expression and TGF-β signaling. It has also recently been shown that PKCλ/ι (PRKC1) has reduced expression in both *de novo* and treatment-induced SCNC.^89^ The authors showed that loss of PRKC1 leads to enhanced mTORC1/ATF4 signaling through phosphorylation of MAPK and LAMTOR2. This change in signaling leads to metabolic re-programming, resulting in increased levels of S-adenosyl methionine (SAM). As this substrate is used for methylation, this change was observed to promote epigenetic changes through DNA hypermethylation and consequently, and induce NED and progression to lethal SCNC. More studies focused on examining the metabolic differences between SCNC and CRPC-adenocarcinoma may reveal additional vulnerabilities that can be therapeutically targeted as this was one of few studies that has attempted to elucidate the metabolic landscape of SCNC and how this might drive the lethal phenotype observed. Another recent report showed that Trop2, a cell surface glycoprotein that is an important regulator of stem-cell renewal, is overexpressed in prostate tumors with NED.^90^ Furthermore, its overexpression in hormone-naïve tumors drives NE marker expression as well as the hyperproliferative phenotype. Further studies showed that Trop2 increased the expression of PARP1 and that Trop2-driven NE tumors were sensitive to PARP1 inhibition. The authors proposed that the Trop2-driven increase in proliferation causes significant DNA damage and inhibition of PARP1 results in apoptosis, representing a possible therapeutic strategy for Trop2 + SCNC tumors.

The aforementioned studies demonstrate NED *in vitro* from luminal-type cell lines. However, it has been recently shown *in vivo* that human basal cells also possess the ability to differentiate into SCNC, when transduced with a combination of oncogenic drivers that increase the proliferative capacity and immortality of the cells. For example, Lee *et al*. demonstrated that human basal cells can be driven to form SCNC tumors by overexpression of N-Myc and myristoylated Akt1 (myrAkt1, partial mimic of *PTEN*-loss), while luminal cells cannot.^91^ Park *et al*. furthermore demonstrated that SCNC genesis can occur when human basal cells are challenged with a combination of factors expressing double negative p53 (TP53DN), myrAkt1, RB1-shRNA, c-Myc, and Bcl2.^92^ As expected, human luminal cells cannot be transformed to SCNC with this same combination of factors. These findings further add complexity to our understanding of SCNC as only luminal and NE cells populate malignant adenocarcinoma glands, where t-SCNC arises.

The biology underlying NED is complex and has only recently been rigorously pursued by the scientific community. Although many mechanisms have been shown to drive SCNC, there still lacks opportunity for therapeutic intervention for many of these mechanisms as most of these proteins are not currently druggable. Furthermore, these results show that there are many possible drivers of NED, which may act individually or collectively in different clinical contexts. Further studies on elucidating the connections amongst the various pathways and opportunities for therapeutic targeting are needed to improve outcomes for patients with advanced disease.

## Conclusions and perspectives

NE cells, despite representing a minor constituent in benign prostate and the vast majority of PCa, likely have important roles in both the physiological maintenance of secretory populations as well as the pathological progression of PCa. As luminal cells are thought to rely on NE cell products, it is conceivable that targeting NE cells (with or without hormone therapy), may represent a promising therapeutic strategy for treating advanced PCa. More studies are needed to further discover NE-specific molecular targets to further validate the therapeutic potential of such a strategy.

**Figure 3.**
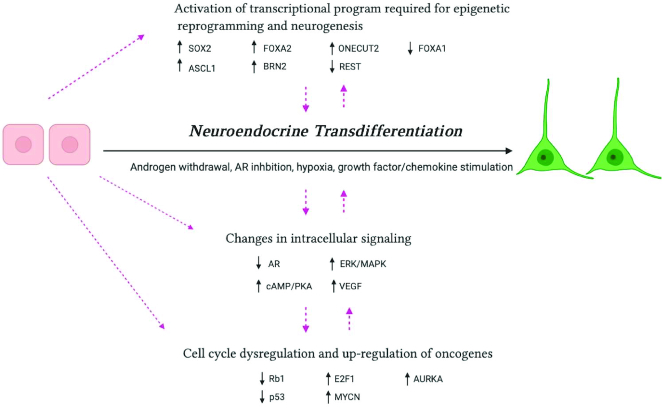
Major reported mechanisms of neuroendocrine differentiation (Created with BioRender.com).

As t-SCNC represents the most lethal course of disease progression, there remains an urgent need to both further define the cell of origin leading to this pathology as well as to discover methods for therapeutic targeting. The vast majority of studies have supported a lineage plasticity theory of transition, suggesting that luminal cells change their cellular identity as a method to evade hormonal therapy. This involves a series of events including expression and repression of several transcription factors to induce epigenetic reprogramming and neurogenesis, alterations in intracellular signaling (which may be a result of or induce the lineage switch), and dysregulation of genes that regulate the cell cycle to induce the classic hyperproliferative, anti-apoptotic phenotype observed in SCNC (Fig. 3). However, nearly all of the studies supporting lineage plasticity as the mechanism of t-SCNC development were performed *in vitro*, which does not represent the complex tumor environment found in humans. Furthermore, as there is significant heterogeneity with human t-SCNC, evidenced by inconsistent expression of NE markers and oncoproteins, it remains entirely possible that both lineage plasticity and clonal expansion of pre-existing NE cells are mechanisms for the development of t-SCNC, depending on the clinical context. Until conclusive studies can convincingly demonstrate whether primary human luminal or NE cells can be transformed into SCNC, the question remains unanswered. Finally, although many mechanisms of NED have been reported, most of these pathways are not currently druggable. It is our belief that more therapeutically vulnerable pathways for SCNC can be discovered through avenues outside of transcriptomic profiling, which is what most research studying SCNC is premised on. This may involve more thorough characterization of cell surface proteins and glycosylation patterns to discover targets that make the cells vulnerable to immunotherapies, probing the intracellular metabolite abundance of SCNC cells to determine preferential metabolic behavior and consequent pathways that can be inhibited, as well as phospho-proteomic studies to discover highly active kinases that can be inhibited to “turn off” preferential signaling pathways. Our understanding of NE cells and NED is only just beginning to become understood and a more thorough understanding of the biological functions and molecular mechanisms of pathogenesis may finally provide curative therapeutic options for men suffering with advanced PCa.
